# Religion: Coping Mechanism or Stressor? A Study of American Male-Factor Infertility Patients

**DOI:** 10.1192/j.eurpsy.2025.295

**Published:** 2025-08-26

**Authors:** A. Garcia Keeme-Sayre, J. Kassab, A. Oppenheimer, M. Duan, J. Huang, B. Stocks, L. Lipshultz

**Affiliations:** 1 Baylor College of Medicine, Houston; 2 Texas Tech University Health Sciences, Lubbock; 3 Urology, Baylor College of Medicine, Houston, United States

## Abstract

**Introduction:**

Seeking treatment for infertility is an emotional process, often provoking symptoms of anxiety and depression in both men and women, especially when treatment fails (Milazzo et al, Plos One; 11). While religion may provide hope and comfort to men with infertility, it may also provoke anguish in those whose religious doctrines restrict certain treatments.

**Objectives:**

The goal of this study is to assess the role of religion in male patients’ ability to cope with their infertility.

**Methods:**

An electronic survey was sent to male patients who presented for an infertility visit at an American urology clinic. Using a 4-point Likert Scale, participants reflected on their religion’s role in seeking treatment for infertility, their selection of treatment, and their ability to cope with their diagnosis. The Likert Scale results were analyzed utilizing T-tests via SPSS Software to assess answer variations across religions. Written responses were analyzed for common themes.

**Results:**

73% of respondents (n=288/395) identified with a religion (58.6% Christian, 29.5% Catholic, 11.8% Other Religion). Across all religions, participants reported that religion served as a significantly greater source of coping than of anxiety in their infertility treatment (2.56 vs. 2.03, t-value = 5.64, p-value = <.00001). In comparison with most other religions, Jewish participants (all non-orthodox) were significantly less likely to report their religion having any effect on their coping at all. Among those written responses that attributed their religion with their ability to cope, the most common key words were “prayer,” “comfort,” and words referring to religious community such as “pastor” (Table 1). Most written responses that indicated their religion had a negative emotional impact stated discomfort with a specific treatment or aspect of the treatment process. Of patients who discussed having disagreed with or gone against their religion’s views (7.9%), the majority were Catholic (n=13/17 (76.5%)).

**Image 1:**

**Figure:**
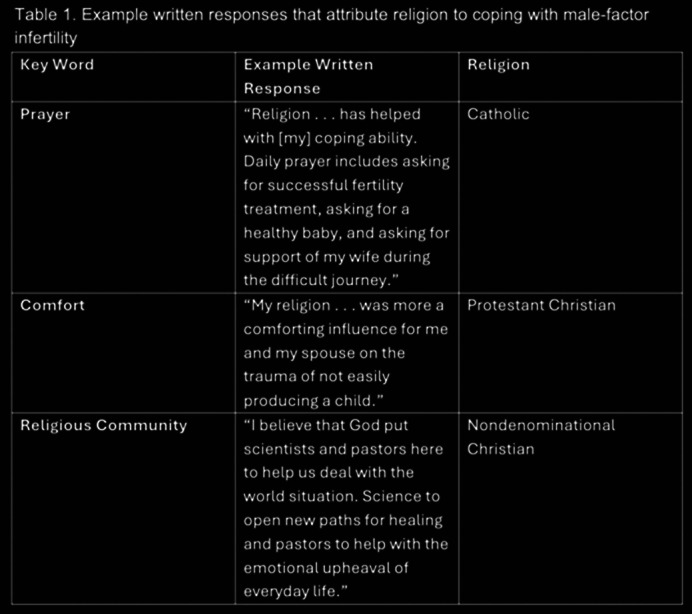

**Conclusions:**

Religion appears to be a source of coping more than anxiety for males with infertility. This is perhaps less applicable to non-orthodox Jewish patients. Providers of male-factor infertility treatment should practice sensitivity when presenting infertility treatment options as a minority of patients may experience cognitive dissonance when considering treatment at odds with their religious identity. However, in religious patients struggling with mental health due to their infertility, physicians can encourage spiritual health and connection with religious community.

**Disclosure of Interest:**

None Declared

